# Therapists and ward staff experiences of virtual reality-assisted aggression treatment in a maximum-security forensic psychiatric clinic

**DOI:** 10.1186/s12888-026-07866-9

**Published:** 2026-02-09

**Authors:** Yrsa Amelie Andersson, Madeleine Leticia Karin Bengtsson, Fernando Renee González Moraga

**Affiliations:** 1 Research Department, Regional Forensic Psychiatric Clinic Växjö, Växjö, Sweden; 2 Department of Psychology, Linnaeus University, Växjö, Sweden; 3 Evidence-Based Forensic Psychiatry, Department of Clinical Sciences Lund, Psychiatry, Lund University, Lund, Sweden; 4 Centre for Ethics, Law and Mental Health, Institute of Neuroscience and Physiology, The Sahlgrenska Academy at the University of Gothenburg, Gothenburg, Sweden

**Keywords:** Virtual reality, VR-assisted interventions, Aggression, Forensic psychiatry, VRAPT

## Abstract

**Background:**

Virtual reality (VR) technology is increasingly being explored as a tool to help reduce aggressive behavior in forensic psychiatric settings. This study aimed to explore the experiences of therapists and ward staff participating in a pilot study of a VR-assisted aggression treatment within a Swedish maximum-security forensic psychiatric clinic.

**Methods:**

A qualitative, descriptive, and exploratory design was employed. Semi-structured interviews were conducted with five therapists and seven ward staff members who participated in the pilot of the newly revised Virtual Reality Aggression Prevention Training (VRAPT). The data, consisting of pre-transcribed interviews, were analyzed using reflexive thematic analysis, carried out separately for each professional group.

**Results:**

The analysis resulted in eight overarching themes. Among therapists, five themes were identified: (1) Navigating treatment in the context of low patient motivation; (2) The perceived value of role-play in VRAPT; (3) Observed patient progress; (4) Structural and procedural barriers to implementation; and (5) Suggestions for future development. Among ward staff, three themes emerged: (1) Perspectives on patient behavior in forensic psychiatry; (2) Experiences of VRAPT in practice; and (3) Perceived opportunities provided by the intervention.

**Conclusions:**

Participants described VRAPT as a promising and engaging method, highlighting its role-play as a particular strength. Nonetheless, technical challenges and contextual limitations were also noted. The study emphasizes the importance of individualizing the intervention to better address patient needs and enhance engagement, therapeutic motivation, and treatment outcomes. By integrating the perspectives of both therapists and ward staff, the findings provide valuable insights into the clinical application of VR-assisted interventions in forensic psychiatric settings.

**Clinical trial number:**

Not applicable.

## Introduction

Forensic psychiatric care in Sweden operates under a dual mandate: to provide care, treatment, and rehabilitation tailored to patients’ psychiatric conditions, while also working to prevent future serious offenses [1–6]. The patient population is highly complex, typically presenting with comorbid psychiatric disorders, psychosocial challenges, and a history of criminal behavior [7]. In Sweden, approximately 300 individuals are annually sentenced to forensic psychiatric care after committing serious crimes, often involving aggressive elements such as assault, homicide, unlawful threats, or arson [8, 9]. These offenses are typically committed under the influence of Severe Mental Disorder (SMD) [10]. Like many other countries, Sweden has legal provisions that address criminal responsibility in the context of mental illness, allowing for diversion from prison to psychiatric care when SMD is present [6, 11]. In cases where there is a significant risk of reoffending due to the underlying mental disorder, the court may impose a Special Discharge Review (SDR) [9]. Under this measure, decisions regarding patient leave or discharge are made by the court rather than the treating psychiatrist, in order to ensure public safety. Research has consistently shown that individuals with SMD have an elevated risk of aggressive behavior compared to the general population [12, 13], while simultaneously being more vulnerable to violent victimization [14, 15].

Aggression remains a prominent issue in forensic settings [16, 17]. Despite a strong emphasis on preventing recidivism, violent incidents during care are not uncommon. Between 2009 and 2017, 13% of forensic psychiatric patients in Sweden were convicted of new crimes while still under compulsory care [4]. Among these, crimes against public authority, particularly violence and threats against officials, were second only to drug-related offenses. It is likely that many more incidents go unreported or are handled internally, as not all criminal acts result in prosecution but may be observed by staff or disclosed by patients themselves [4]. Aggression is particularly prevalent in inpatient settings, especially among patients subject to SDR. In many cases, this violence is directed toward staff members, though other patients may also be affected [4.11]. To address these risks, forensic psychiatric care includes various interventions such as pharmacological treatment, structured daily routines, psychoeducation, and psychosocial support. However, clinicians must continuously navigate the tension between meeting therapeutic needs and ensuring safety for staff, patients, and society at large [11, 18].

Scientific evidence supporting psychological and psychosocial interventions in forensic psychiatry remains limited. Significant knowledge gaps exist, particularly regarding the effectiveness of treatment approaches in this population [19–21]. A notable lack of high-quality randomized controlled trials (RCTs) contributes to this gap [19]. This does not imply that current treatments lack value; rather, it highlights the need for more rigorous research to evaluate their outcomes [19–21]. Moreover, many therapeutic methods in use have been developed for general psychiatric populations or voluntary care settings. Their applicability to involuntary forensic settings, where patients frequently present with complex, overlapping diagnoses, may therefore be limited [20]. In this context, the need for research into interventions specifically targeting violent behavior has been underscored as a priority [21].

Aggression can be described as a behavior aimed at harming others [22, 23]. It imposes a substantial burden on societies worldwide, generating costs related to psychiatric care, physical injuries to victims, sick leave, police interventions, and various associated services [24, 25]. Within forensic psychiatry, aggressive incidents are among the most harmful and challenging, as they can result in serious physical or psychological harm to both fellow patients and staff members [26]. Such incidents can contribute to an unsafe and stressful environment, further elevating anxiety among patients and healthcare providers. Patients who display aggressive behavior are often subjected to increased isolation or transferred to higher-security units, measures that may hinder their treatment and rehabilitation process [27]. Instrumental (proactive) aggression is goal-directed behavior used as a means to an end (e.g., coercion or resource acquisition), whereas reactive (impulsive) aggression is an affect-laden response to perceived provocation or threat; importantly, these functions can co-occur and are best treated as dimensions rather than mutually exclusive types, while psychotic aggression refers to aggression arising from psychotic symptoms (e.g., delusions or hallucinations) rather than goal-directed or affective processes [28–30]. The triggers for aggression vary widely across individuals, but frustration is a common cause, particularly among those with a tendency toward reactive aggression or individuals with antisocial personality disorder [31, 32]. Aggression can be explained through various psychological models, including Social Learning Theory [33] and the Frustration-Aggression Hypothesis [34]. However, this study focuses on two specific models: the Social Information Processing (SIP) Model [35, 36] and the General Aggression Model (GAM) [23, 37, 38], as they form the theoretical foundations of VRAPT. The first version of VRAPT was built upon the SIP model, while the revised and current version is based on GAM.

The GAM was developed to integrate earlier theories such as social learning theory and cognitive script theory into a comprehensive framework [23, 37, 38]. GAM is often described as a biosocial-cognitive model, as it considers how biological predispositions, individual traits, and environmental factors interact to influence aggressive behavior [23, 37–39]. Biological factors may include elevated stress hormone levels or impulsivity, such as that observed in ADHD, which might increase sensitivity to provocation [39]. Personal traits, such as temperament, life experiences, and learned response patterns, can either amplify or mitigate the influence of situational triggers [38, 39]. Environmental factors refer to immediate situational inputs like loud noises, emotional intensity in one’s surroundings, crowding, or other provocative stimuli that may elevate stress levels and lead to aggressive responses [38, 39]. According to GAM, these interacting factors shape how an individual perceives, interprets, and reacts to a given event, thereby influencing whether the outcome will be aggressive or not [39]. The model also emphasizes the role of learning in shaping future behavior: each situation creates new experiences that are encoded and gradually form automatic response patterns, which then guide the individual’s interpretation and reactions in subsequent situations. This cyclical nature is a defining feature of the GAM framework [23, 39]. As such, GAM provides a useful conceptual basis for understanding both instrumental and reactive aggression, offering a nuanced framework that helps to inform and improve aggression-focused interventions by recognizing the multifactorial nature of aggressive behavior [38, 39].

Virtual Reality Aggression Prevention Training (VRAPT) is an innovative treatment method developed in the Netherlands, aimed at reducing aggressive behavior among forensic psychiatric patients through the use of Virtual Reality (VR) [40]. The patient is immersed in a virtual reality by means of goggles with a display depicting controllable environments in which they are able to move around as an avatar. Other avatars in this environment are controlled by the therapist. The patient also wears headphones, cancelling out noise from the actual surroundings while emitting sounds from the virtual surroundings, including the distorted voice of the therapist, in the character of their avatar. An example of such a virtual scene might be a post office, where the patient is asked to pick up a package, with the therapist playing the role of the clerk. The therapist might choose to not understand the patient, or be uninterested or show any other number of behavior that may aggravate the patient, allowing them to practice controlling their aggression in a triggering situation. These environments are tailored to each individual, as much as the software allows. Stressors applied in VR have been shown to elicit similar stress responses as compared to physical reality stressors [41, 42]. Immersion is a term often used in relation to VR and is meant to illustrate how a system can help a person shut out reality, which it does best if it offers multiple sensory inputs, coordinates the individuals’ movements to that of the avatar, and shuts out the external world [43]. VR technology enables the elicitation of psychological, physiological, and behavioral responses to aggression in a safe and controlled environment, allowing patients to explore and rehearse adaptive behaviors to manage their own and others’ aggression. Within VRAPT, patients engage in social interactions with virtual characters and practice recognizing others’ facial expressions and regulating their own and others’ aggressive impulses in realistic scenarios [40]. Therapists are able to control the virtual environments and avatars, tailoring the experience to the individual needs of each patient [44]. Examples of virtual environments include a café, a park, a bus, and a convenience store. The therapist can also manipulate avatar features such as facial expressions, voices, movements, and verbal responses using voice distortion technology [45].

The VRAPT was grounded in the SIP model, which is frequently used to explain reactive aggression in children and adolescents [35]. According to the SIP model, aggressive responses result from deficits in interpreting and processing social information. The model outlines six cognitive steps: encoding social cues, interpreting those cues, clarifying goals, generating possible responses, selecting a response, and enacting the behavior. These steps initially formed the basis for VRAPT session content [35, 40]. The first randomized controlled trial (RCT) evaluating VRAPT in a forensic psychiatric setting in the Netherlands found no significant reductions in aggression compared to a waitlist control [44]. The study included 128 patients, with a dropout rate of 20%, which is consistent with similar psychological treatment trials in inpatient settings. Although some secondary outcomes, such as hostility and anger management, showed positive effects, these gains were not maintained at the three-month follow-up [44]. Following the RCT, the VRAPT manual was revised [45, 46] and pilot studies were subsequently conducted in both Swedish forensic psychiatry and correctional services [45]. The revised version is now conceptualized and manualized according to Cognitive Behavioral Therapy (CBT) principles, and its theoretical foundation has been expanded to include not only the SIP model but also the GAM. Whereas the SIP model emphasizes cognitive processes in response to specific social situations [35], the GAM offers a broader, cyclical understanding of aggression that incorporates cognitive, emotional, and contextual variables [23]. A Dutch pilot found that theoretical framework with the SIP model was too hard to grasp for the participants [47]. In this study we used the VRAPT model, a simplified version of the GAM to illustrate the conceptual framework of the current VRAPT [45]. This shift means that sessions are no longer structured around the six discrete SIP stages but instead emphasize broader individual and contextual vulnerabilities [45, 48]. In line with this development, the revised VRAPT moves beyond a solely cognitive focus by broadening its scope to include thoughts, emotions, and learned patterns of aggressive responses over time. This approach reflects a biopsychosocial-cognitive framework that complements the GAM model, underlining both individual and contextual vulnerabilities as well as the roles of emotion recognition and regulation in the etiology and treatment of aggression.

VRAPT consists of 16 sessions divided into four modules [45, 46, 48]. In the initial sessions, the patient’s dysfunctional aggression is conceptualized, and the VR system is introduced along with an opportunity to explore the virtual environments. In the subsequent sessions, patients practice identifying and differentiating emotions, both in themselves and in virtual avatars, during aggression-provoking role-plays. The third module emphasizes skills training for managing personal and interpersonal aggression through continued VR-based role-play. The final session involves treatment evaluation and a comprehensive summary of progress [45]. Therapists also had access to physiological monitoring equipment that measured heart rate variability (HRV) and skin conductance during parts of Module B and consistently throughout Module C. These physiological markers were used to provide feedback to therapists on patients’ levels of physiological arousal during role-play exercises [45, 46]. In subsequent interviews and qualitative analyses, this equipment was referred to by patients and staff as the pulse watch.

A pilot study investigating the effects of VRAPT on violent offenders within the Swedish Prison and Probation Service found that 42% of participants (6 out of 14) showed improvements in aggression. Emotional regulation improved for 65% (9 out of 14), and 71% (10 out of 14) demonstrated better anger management [49]. It is important to note, however, that four participants (29%) exhibited deterioration in at least one outcome measure, although no individual worsened across all measures. A common factor among those who showed deterioration was higher self-reported levels of emotional and physical neglect. Based on this, the authors suggest that offenders with such backgrounds may not be the most suitable candidates for VRAPT. While the small sample size and lack of a control group limit the generalizability of the findings, the authors nonetheless highlight the potential of VRAPT to positively influence aggression, emotional regulation, and anger management. They also emphasize the need for further research, particularly to identify which subgroups of offenders are most likely to benefit from VRAPT and to explore potential risks associated with its use [49].

In a qualitative sub-study within Swedish forensic psychiatry, patients’ experiences of VRAPT were examined as part of a larger pilot project [48]. An inductive manifest content analysis was conducted, resulting in six content categories: (1) The therapeutic process, (2) The VRAPT method, (3) VR technology, (4) Previous treatment experiences, (5) Challenges in the treatment of aggression, and (6) Unexpected experiences. The study captures the patients’ nuanced experiences of the intervention. For example, skills training through VR role-play was appreciated and perceived as beneficial by several patients. Although some appreciated the structure of the treatment, many found the homework assignments repetitive and frustrating. Some also felt that treatment goals were unclear or missing [48]. A lack of motivation to address one’s aggression was identified as a treatment barrier for certain patients, with some attempting to manipulate the intervention by saying what they thought the therapist wanted to hear [48]. While some participants responded positively to VR technology, others found that limitations in graphics and movement within the virtual environment reduced the sense of realism. Patients’ perceptions of their learning and skill development following VRAPT varied: some reported that the program helped them manage their aggression, while others felt that they lacked sufficient strategies once the intervention ended. Nevertheless, many patients described how VRAPT helped them identify personal strengths and weaknesses, which in turn enhanced their self-confidence, self-awareness, and capacity for reflection. Communication, a sense of safety, and a strong therapeutic alliance were frequently emphasized as crucial elements of the treatment process. Suggestions for improvement included more individualized virtual scenarios and extended opportunities to practice within sessions and over a longer treatment period [48].

The aim of the present study is to contribute to the evaluation of the VRAPT intervention. Specifically, the authors seek to explore and understand how therapists and ward staff within forensic psychiatric settings experience VRAPT and its implementation, in order to capture the perceived effects and implications of the method from multiple professional perspectives. The findings will serve as a foundation for the future development of VRAPT. While the primary audience for this study consists of professionals working in forensic psychiatry, it may also be of interest to other practitioners engaged in aggression management or those curious about the clinical application of virtual reality technologies. One of the overarching goals of VRAPT is to contribute to a safer environment for both patients and staff in forensic psychiatric care. While patients’ experiences have been addressed in a previous article [48], the current study aims to highlight the perspectives of therapists and ward staff, thereby contributing to a more comprehensive understanding of VRAPT by adding new insights. Given the descriptive and exploratory nature of this study, its aim is to investigate and interpret a relatively new research domain [50]. The findings should not be viewed as objective representations but rather as analytically generated insights that illuminate patterns in participants’ experiences. This background has led to the following research questions: How do therapists and ward staff experience Virtual Reality Aggression Prevention Training (VRAPT) and its implementation within forensic psychiatric care?, What changes do therapists and ward staff observe in patients following the start of VRAPT?, What are the perspectives of therapists and staff regarding the further development of VRAPT?, What similarities and differences can be identified between therapists’ and ward staff’s accounts of their experiences with VRAPT?

## Method

### Research design

This qualitative study is a language-based analysis in which the results are presented in textual form [50], based on transcribed interviews conducted by a third party. The study has an exploratory character, as it aims to investigate a novel area and generate relevant questions for future development of VRAPT [50]. The study employs Reflexive Thematic Analysis [51]. Reflexivity in this approach entails the researchers’ continuous reflection on their own assumptions, prior experiences, knowledge, biases, interpretations, and emotions that may influence the analytical process [51]. Two separate thematic analyses were conducted, as the interviews with each professional group (therapists and ward staff) were based on distinct sets of interview questions. Combining all interviews into a single analysis would have risked diluting the thematic structure due to the broad range of perspectives and experiences. Analyzing each group separately allowed for clearer identification and comparison of themes, enabling a more nuanced understanding of how VRAPT was perceived and implemented.

This study applies a hermeneutic perspective, meaning that the researchers actively examine how their pre-understanding of the topic may influence the interpretation of the material [50]. For instance, codes in the analysis are not seen as representations of an objective truth, but rather as tools for understanding the participants’ narratives, reflecting the hermeneutic stance embedded in the analytical approach [51]. The study is grounded in critical realism, which assumes that an underlying reality exists, but that it cannot be fully captured due to the influence of interpretation and context [51]. Since human beings are subjective, and knowledge is always a temporary interpretation, understandings may vary depending on personal development and newly acquired insights [51]. As such, this study operates under a realist ontology, while acknowledging an epistemology rooted in relativism. In the context of reflexive thematic analysis, this means the authors do not aim to present objective truths, but rather interpretations of truths, subjective insights into individuals’ experiences. Themes are therefore developed through active engagement with the data, rather than through pre-existing coding frameworks.

### Participants

The study included five therapists and seven ward staff members from the participating forensic psychiatric clinic. All therapists were either licensed psychologists or certified CBT therapists who had completed 16 h of VRAPT-specific training prior to administering the intervention. In addition, the therapists received continuous supervision throughout the treatment process from the research group who adapted the VRAPT method in Sweden. The ward staff, those who interacted most closely with patients in the day-to-day ward environment, were involved in the intervention with the patient’s consent, primarily to support tasks such as completing assessments and filling out treatment-related forms. It is likely that the participants’ perceptions of VRAPT were influenced by their professional backgrounds. The therapists’ views may have been shaped by their clinical experience with psychological treatment, whereas the ward staff’s perspectives were more likely grounded in their day-to-day interactions with patients.

### Ethical considerations

This study and informed consent were approved by the Swedish Ethical Review Authority (Dnr: 2019–02337; 2020–06317), and all participants provided their written consent to participate in the study. Ethical sensitivity was especially important given the setting, a maximum-security forensic psychiatric clinic, and the nature of the participants’ roles. Ward staff, who maintain direct and continuous contact with patients, hold a duty of care toward a highly vulnerable population. Therapists, while working within the same institution, were not involved in any other treatment of the patients enrolled in the intervention. This role distinction helped mitigate some aspects of the dual-role dilemma, in which clinical and research responsibilities may conflict. Nevertheless, ethical risks remained, particularly the potential for indirect instrumentalization of patients when staff contribute to research on interventions implemented in their own workplace.

The primary ethical considerations in the present study focused on the analysis and interpretation of the material. Key concerns included ensuring confidentiality, maintaining data integrity, and avoiding bias, such as selectively reporting only positive or negative experiences of the intervention. All participants provided written informed consent prior to the interviews. The authors engaged in continuous dialogue with the principal investigator to ensure that both favorable and critical reflections on the intervention were treated as equally valuable to the broader research aims. This approach supported a balanced and honest interpretation of the data.

To safeguard participant confidentiality, all interview transcripts were transferred via USB rather than online and stored locally on password-protected personal computers. By capturing the perspectives of professionals working within this unique clinical context, the study contributes ethically grounded insights that can inform the future development of VR-based interventions, ultimately promoting safer and more therapeutic environments for both staff and patients.

### Recruitment process

The study recruited both therapists who had delivered VRAPT and ward staff who had worked closely with patients participating in the intervention. Potential participants were informed about the study both verbally and in writing by independent research personnel. The written information outlined the purpose of the study, the procedure, and confidentiality measures. It also emphasized that participation was entirely voluntary, that participants could withdraw at any time without providing a reason, and that non-participation would not affect their employment status or working conditions. No financial or other compensation was offered for participation, and the study was not associated with any risks to participants. After receiving the information, participants were given approximately one week to consider their involvement and were offered the opportunity to contact the research group with any questions. Written informed consent was obtained, and all data was handled confidentially. All therapists and ward staff invited to participate provided their consent. It is important to note, however, that only those ward staff who were responsible for patient evaluations were interviewed.

### Sampling strategy

The study employed purposive sampling, a non-random sampling technique commonly used in qualitative research [52, 53]. In purposive sampling, participants are deliberately selected based on predefined criteria relevant to the aims and objectives of the study [52]. This approach typically involves recruiting individuals or groups with specific knowledge or experience related to the phenomenon being studied [53]. Purposive sampling is considered both time- and cost-effective, as it prioritizes participants who are most likely to provide meaningful, rich, and nuanced data, critical elements in qualitative research [52]. Several types of purposive sampling have been identified [52–54]. This study used a combination of criterion sampling and maximum variation sampling. In criterion sampling, participants are selected based on specific, predefined criteria aligned with the research objectives [52, 53]. In this case, eligible participants were either therapists who had delivered the VRAPT intervention or ward staff who had worked closely with the patients during the treatment process. Maximum variation sampling, also known as heterogeneous purposive sampling, was used to capture a range of perspectives by including participants with diverse characteristics [52]. By involving both therapists and ward staff, the study aimed to provide a more comprehensive and multifaceted understanding of experiences with VRAPT from two distinct professional viewpoints.

### Data collection

As the aim of this study was to explore and understand staff members’ subjective experiences of the VRAPT intervention, a method that allows for in-depth and nuanced responses was required. A qualitative approach, in particular, the use of interviews, was therefore deemed appropriate. The semi-structured interviews conducted in the project were based on a set of predefined questions, while also allowing flexibility for follow-up questions to be asked spontaneously as the conversation unfolded [50]. The interviews were conducted by individuals who had no prior professional or personal relationship with the participants, in order to minimize potential bias in the responses. All interviews were carried out after the VRAPT intervention had concluded and lasted approximately 45 min each. The questions were open-ended and focused on topics such as how staff experienced various aspects of VRAPT, their attitudes toward VR prior to the program, and their general views on the use of VR in forensic psychiatric settings. To provide the reader with a clearer sense of the focus and differences between the two interview guides, one or two example questions are provided. For therapists, the questions were more detailed and treatment-specific, addressing their direct experience of VRAPT (e.g., how they experienced the VRAPT method as clinicians, which components participants appeared to benefit from most or least, and how supporting materials such as workbooks were used in practice). For ward staff, whose involvement was more indirect, the questions were broader and focused on their role in the participant’s VRAPT process and everyday care (e.g., how they were involved in the participant’s work with VRAPT, their awareness of treatment goals, and reflections on how these goals were addressed and followed up in daily ward routines). All interviews were audio recorded and subsequently deleted after transcription. The authors (YA, MB) were granted access to the anonymized transcripts for the purpose of conducting the present study.

### Analysis

As previously stated, this study employed Reflexive Thematic Analysis (RTA) [51]. The aim of this method is not to uncover an objective truth, but rather to offer an interpretative analysis of participants’ experiences and perceptions. In RTA, the researcher plays an active role in constructing themes, rather than simply identifying them as inherent in the data. The process is iterative, flexible, and acknowledges subjectivity that is inherent in qualitative research. This analysis followed an inductive approach, meaning that theoretical insights emerged from the researchers’ engagement with the data itself [51], and allowed for the possibility of adapting the research questions in response to insights developed during the analytic process [50]. Semantic coding refers to interpreting the text at a more surface level, staying close to what is explicitly stated [51]. Latent coding, by contrast, involves interpreting underlying meanings that are not directly articulated in the text but are inferred to lie beneath the surface. These are not mutually exclusive modes of interpretation but rather points on a continuum, with the understanding that different codes may fall at different positions along this spectrum [51].

Initially, the authors expected that the analysis would primarily remain at the semantic level. However, it soon became evident during the coding process that although the authors (YA, MB) had neither been present during the interviews nor had access to the audio recordings (due to confidentiality protocols), certain passages required latent interpretation. This was due to the natural flow of spoken language: participants’ responses often emerged as they recalled memories spontaneously, which did not always follow a linear or structured format typical of written communication. Furthermore, interviewees did not always respond directly to the questions asked, instead offering other relevant experiences and reflections not explicitly prompted by the interviewer. As a result, participant quotes could not always be interpreted literally as direct answers to specific questions. This required the authors to occasionally apply a deeper, more interpretative lens in order to understand the intended meaning and the underlying message. Solely using semantic interpretation would have risked missing important nuances in what participants were actually trying to convey. Therefore, the authors drew upon the full range of interpretive depth along the semantic–latent continuum. Coding was conducted without narrowly focusing on the predefined research questions, although it was acknowledged that the interviews broadly addressed the core topics of interest. During theme development, ongoing checks were made to ensure that the themes remained relevant and aligned with the study’s research questions.

### Data analytic strategies

The authors followed the six-phase process of thematic analysis [51]. All six phases were carried out twice, once for each interview group. Phase one involved familiarization with the data through repeated reading of the transcripts (or other forms in which the data were presented) to develop a deep understanding and to begin identifying patterns [51]. In this study, the authors each read the transcribed interviews twice. During the first reading, informal notes and “doodles” were made on paper to begin processing the content intuitively [51]. The second reading was done with digital comments in Word, where emerging reflections and questions were noted. Phase two focused on generating initial codes. The first interview was coded jointly by both authors to establish a shared understanding of the coding process. The remaining interviews were then divided between the authors for independent coding, using a shared document that allowed ongoing visibility of each other’s work and continuous communication. No specialized software for thematic analysis was used. Instead, the coding was carried out using the comment function in Word. As this was an inductive process, codes were not developed a priori but emerged from the data itself. Phase three involved the development of preliminary themes by identifying patterns among the codes and clustering them accordingly [51]. The authors compiled a list of all codes and systematically reviewed them, organizing them into groups using a table format. This stage also included discussions about emerging questions and uncertainties from the coding phase. In phase four, initial themes were reviewed and refined, and their relevance to the research questions were evaluated. In phase five, the themes were finalized and named. These phases are not strictly linear but iterative and recursive, the process often involves moving back and forth between phases [51]. This process resulted in five main themes and 14 subthemes for the therapist group, and three main themes and six subthemes for the ward staff group, all of which were, in various ways, relevant to the study’s research questions. See Tables 1 and 2 for examples of finalized themes, subthemes, codes, and supporting quotes. In phase six, the writing process began [51]. Short summaries of the codes within each subtheme (or within the main theme if no subthemes existed) served as the basis for descriptive texts. These were supported by illustrative quotes from the transcripts, grounding the authors’ interpretations in the participants’ own words.

**Table 1:** Example of the analytical process from quote to Code, Subtheme, and theme (Therapist Interviews)

Quote	Code	Subtheme	Theme
“… because as it stands, we can’t adapt the homework. We have no choice—it’s the same task we have to give to the patient. If it doesn’t work, we don’t have an alternative, and that’s where we lost a lot. So more could be done there.”	Homework tasks perceived as unchangeable	Challenges related to goals and homework tasks in workbook	When treatment conflicts with low motivation

**Table 2:** Example of the analytical process from quote to Code, Subtheme, and theme (Ward staff Interviews)

Quote	Code	Subtheme	Theme
“I would describe him as one of the most violence-prone patients in the entire clinic, with a history of extremely brutal violence. On the ward, he’s always been very open about his thoughts of harming people—this was already the case before VRAPT. He would often talk about it and thought a lot about hurting others, pretty much every day. So before, there was a lot of violence, irritation, and he was easily provoked.”	Violence-prone patient with extensive history of aggression	Patients’ everyday behavior prior to VRAPT	Experiences of patients in forensic psychiatry

### Methodological integrity

As the transcripts were provided to the authors in anonymized form, there was no possibility of identifying the interview participants. This ensured the protection of anonymity and participant integrity. A total of 12 interviews, six with therapists and six with ward staff, were used for two separate thematic analyses, which were later compared in the discussion. This approach deviates slightly from conventional qualitative analysis, which typically involves a single dataset. However, this design was chosen both to ensure sufficient data volume and to allow for the exploration of differing and overlapping perspectives on VRAPT from two distinct professional groups. Analyzing only one group would risk overlooking valuable insights. A third perspective, patients’ experiences of VRAPT, has been presented in a previous publication [48], and these findings are also considered in the discussion of the present study. The authors employed triangulation at multiple stages to enhance the study’s reliability. This includes comparing perspectives across professional groups in an effort to present an honest and nuanced picture of VRAPT. Another form of triangulation occurred during coding and theme development, where both authors were involved in reviewing all interviews to avoid the emergence of a singular interpretative path. Throughout the process, the authors maintained a logbook, took notes, and engaged in frequent and structured written and verbal communication. This was done to ensure transparency and reflexivity, and to document key decisions, ideas, and interpretations as the study progressed. ChatGPT was used solely to clarify methodological questions, assist with translation, including facilitating the initial draft translation from Swedish to English, and to suggest synonyms or wording alternatives. It was not used to interpret the data or to generate substantial portions of the manuscript.

## Results

The aim of this results section is to highlight relevant content from the interviews with therapists and ward staff involved in the project, in order to address the four research questions and contribute to the overall evaluation of VRAPT. As the two groups had different levels of involvement in the intervention and were therefore asked slightly different interview questions, the analysis and results are presented in two separate parts. Our analysis of the therapist interviews resulted in five main themes and 14 subthemes (see Table 3). The analysis of the ward staff interviews produced three main themes and six subthemes (see Table 4).

### Therapist interviews

**Table 3:** Summary of results from interviews with therapists

Main Theme	Subtheme
When Treatment Conflicts with Low Motivation	Challenges in Collaboration and Motivation
	Challenges Related to Goals and Homework Tasks in Workbook
The Importance of Role-Play in VRAPT	Role-Play as a Key to Engagement and Emotional Activation
	VR Enhances Safety and Immersion in Role-Play
Experiences of Patient Development	Insight and Increased Self-Understanding
	Applied Strategies and Behavioral Change
Barriers and Challenges to Treatment Delivery	Technical Issues and Equipment Limitations
	Difficulties with inflexible treatment materials
	Lack of Time and Space
	Challenges Related to the Complexity of the Patient Group
Ideas for Further Development	Technical Improvements
	Adaptation of Materials
	Development and Revision of the Treatment Program
	Strengths and Opportunities of VRAPT

### Theme: When treatment conflicts with low motivation

This theme focuses on therapists’ experiences of working with VRAPT in cases where patient motivation was lacking. It captures recurring challenges in the therapeutic process and is divided into two subthemes: Challenges in Collaboration and Motivation and Challenges Related to Goals and Homework Tasks in Workbook.

#### Subtheme: Challenges in collaboration and motivation

Therapists reported that many patients demonstrated a lack of motivation and engagement, which constituted a major barrier to successful treatment. This lack of motivation was often attributed to patients perceiving the content as unrelated to their own issues. One therapist reflected on how VRAPT struggled to address instrumental violence, which in turn affected motivation among patients with such behavioral patterns:

… since it was more instrumental [violence] than impulsive, it makes it difficult to evaluate the method itself. You don’t know whether it’s the method that feels off or the whole situation.

Another reason for low motivation, according to the therapists, was that some patients found the exercises “boring” and had expected the VR component to be more entertaining, an expectation that initially attracted them to participate in the program.

The therapeutic alliance also faced challenges when patients tried to present themselves in an overly favorable light and found it difficult to open up emotionally. In contrast, patients with previous experience of psychological treatment, such as CBT, were perceived as more engaged and aware of their behaviors. As one therapist explained:

So [they] were significantly more engaged. [They] had gone through CBT treatment before, focusing on impulse control and such, and it was evident that [they] were aware of their own behaviors and problems, [they] had a much better understanding of which situations triggered [them] and were much more open. So I believe that treatment was more successful.

#### Subtheme: Challenges related to goals and homework tasks in workbook

Therapists noted that several patients struggled with completing homework tasks and engaging in goal-setting. One therapist commented on the rigidity of the existing goal structure in the VRAPT program:

What I notice when talking to other therapists is that everyone ends up with the same treatment goals because VRAPT has predefined goals.

Some therapists perceived the fixed goals as a limitation to the therapeutic process. Others emphasized the importance of having clear goals, especially for this patient group, since they may not always know what they need. Although the repetitive nature of the goals across sessions was acknowledged, it was generally viewed as helpful:

… it was there and repeated in every chapter, every session. It followed a sequence in the manual. So in that sense, it was appreciated, even if it was repetitive, it served a purpose.

Therapists also expressed a desire for the homework tasks to be more adaptable in order to increase their relevance and meaning for each individual patient:

… because as it stands, we can’t adapt the homework. We have no choice—it’s the same task we have to give to the patient. If it doesn’t work, we don’t have an alternative, and that’s where we lost a lot. So more could be done there.

### Theme: The importance of Role-Play in VRAPT

Role-play was frequently mentioned in the interviews and was consistently described as a valued component of the VRAPT intervention. Therapists considered role-play to be a generally beneficial therapeutic tool and noted that virtual reality added an important dimension by enhancing immersion through visual cues and voice distortion. This theme is divided into two subthemes: Role-Play as a Key to Engagement and Emotional Activation and VR Enhances Safety and Immersion in Role-Play.

#### Subtheme: Role-Play as a key to engagement and emotional activation

Therapists identified role-play as a central methodological component of VRAPT. Several therapists and patients appreciated the VR-based role-plays because they enabled concrete and effective learning. The immersive VR experience also appeared to increase patient engagement, as described by one therapist:

… it was also a fun element for our patients, because almost all of them enjoy computers and apps… they become more active in the treatment instead of just sitting and talking… and they remember it better that way.

#### Subtheme: VR enhances safety and immersion in Role-Play

Therapists highlighted several advantages of conducting role-play in VR. VR-based scenarios allowed for the recreation of high-risk situations in a safer and more controlled manner compared to in vivo role-play. In a forensic psychiatric setting, the opportunities to rehearse different real-life situations are limited, making it difficult to simulate emotionally charged or dangerous scenarios in the physical environment. VR, however, made such simulations feasible and practical. One therapist explained:

The biggest advantage of VR is that we can’t really provoke the most intense or dangerous situations in real life… But in VR, you can come up with any scenario depending on the patient’s triggers, when it comes to their anger, violence, or similar issues.

Another benefit of VR-based role-play mentioned by several therapists was the enhanced sense of realism and emotional engagement. According to participants, it was easier for both therapists and patients to become immersed in the role, allowing for more authentic emotional responses. As one therapist put it:

… it becomes easier for both me and the patient… I get into it more, the patient gets into it more, and we both know it’s a situation we’re pretending is happening—and it’s easier to pretend when you also have visual support from the VR.

### Theme: Experiences of patient development

In the interviews, therapists described how patients appeared to have developed in various ways during the VRAPT intervention. This included both internal psychological growth and observable behavioral changes in everyday situations. Two subthemes reflect the therapists’ perceptions of patient development: Insight and Increased Self-Understanding, and Applied Strategies and Behavioral Change.

#### Subtheme: Insight and increased self-understanding

Several therapists noted that, throughout VRAPT, patients began to reflect on their emotions and reactions, which led to greater self-awareness. One therapist described their patient as follows:

…[they] learned something about themselves, that they don’t react to situations the way they thought they would.

Therapists also observed that patients gained a better understanding of the interaction between their own and others’ emotions and behaviors, as well as insights into what triggered their anger:

… it’s much easier to be aggressive when someone else is being aggressive back. That kind of insight is something we worked on a lot.

#### Subtheme: Applied strategies and behavioral change

Some therapists reported that patients developed and improved practical skills such as problem-solving, communication, and impulse control, which they began to apply in daily life. One therapist explained how their patient learned to pause and reflect rather than act impulsively:

… it was almost unconscious, but [they] just… it happened automatically. When something happened on the ward, [they] started thinking about certain strategies.

In some cases, patients were described as applying alternative strategies, for example, using assertive communication instead of aggressive behavior:

… [the patient] could speak up to the doctor in a way they hadn’t done before—not in an aggressive way, but more in a confident manner, expressing what they thought and felt.

### Theme: Barriers and challenges to treatment delivery

Various challenges related to the VRAPT intervention were frequently mentioned during the interviews. The most prominent issues have been categorized into four subthemes: Technical Issues and Equipment Limitations, Difficulties with Inflexible Treatment Materials, Lack of Time and Space, and Challenges Related to the Complexity of the Patient Group.

#### Subtheme: Technical issues and equipment limitations

A significant number of statements concerned technical malfunctions and equipment that failed to function as intended. A wrist device designed to measure physiological data such as heart rate and skin conductance was repeatedly mentioned as never working properly. Another frequently noted issue was the cognitive overload therapists experienced when managing multiple components simultaneously, which impaired the effectiveness of their work:

I found it challenging—you need four separate screens to control at once, while also observing the patient and engaging in conversation. So, it ended up being five things at the same time, plus controlling the avatars in VRAPT, it’s not exactly easy.

Therapists also found the software to be restrictive, particularly in terms of the limited number of environments and non-verbal interactions available. One therapist explained:

For instance, you were still quite limited in what you could do in the VR environment. There were only a certain number of gestures and a certain number of settings you could access, and you became increasingly aware of these limitations over time.

Some therapists commented that the graphic quality was below expectations, especially during the exercise where patients were asked to interpret facial expressions. Although this component was seen as promising, it ultimately did not meet expectations:

… the facial recognition part didn’t really work. I remember that the two patients who dropped out did so right after we completed that part. They complained that it was boring and that it took too much time.

#### Subtheme: Difficulties with inflexible treatment materials

Therapists expressed that the program materials were not adaptable to individual needs. The materials contained a large amount of repetitive text, which was experienced as difficult for this patient group. One therapist described how this limited their ability to follow the program as intended:

I think it was… they’re not used to working in that way, to take responsibility for a workbook. So we didn’t really use it, the patient’s part… It worked well in the beginning, when they were observing facial expressions in everyday life and so on, but then around session 8, when the same session repeats over and over, it stopped working.

Several therapists also noted that motivating patients to complete the homework was challenging. One therapist described how the sheer volume of questions and the size of the workbook could be overwhelming:

Those homework assignments in their book, it was hard to get patients motivated to fill it out. There were too many questions, the book was too thick. Just showing it to them, it was almost a shock. Many just backed away. They don’t have the capacity, so to speak, to handle that kind of task.

#### Subtheme: Lack of time and space

Many therapists felt that the manual contained more material than could realistically be covered in a single session. Several interviewees discussed the fast pace of the treatment, and the insufficient time allocated per session:

I rarely managed to complete the sessions as written in the manual, time-wise. Even though my sessions were at least twice as long, 90 min, we still didn’t have time to go through the new scenarios. It just didn’t happen.

Therapists also expressed frustration about the lack of space for spontaneous conversations about real-life events affecting patients between sessions. They wished for more flexibility to address these issues when they arose:

… the patient needed to talk about things that had happened between sessions, but there was no room for that. The manual is quite rigid.

#### Subtheme: Challenges related to the complexity of the patient group

Therapists noted that the method was not equally suitable for all patients or diagnoses. Individual characteristics strongly influenced engagement and outcomes. ADHD was frequently cited. One therapist explained:

So, the first patient had an ADHD diagnosis, which ruined it because they couldn’t tolerate the repetitive nature when we went over the structure of the treatment.

The type of aggression that VRAPT is designed to target was also discussed. Several therapists noted that the program was more suited for treating reactive aggression and not instrumental violence:

… the patient was mismatched with the treatment method. The intervention is more suited to reactive aggression, but this patient displayed more instrumental violence… So maybe that’s why, it was basically impossible to carry out the sessions.

In relation to instrumental aggression and antisocial personality disorder, which are common among forensic psychiatric patients, another therapist stated:

The problem was that this particular patient had no intention of stopping their violent behavior. So, they weren’t a good fit for the treatment, we couldn’t even set that as a goal, because the patient didn’t want to stop doing what they were doing.

Cognitive impairments and borderline intellectual disabilities (ID) were also described as barriers that made it difficult to follow the manualized treatment structure.

### Theme: Ideas for further development

This theme explores therapists’ reflections on what worked well in VRAPT and their suggestions for improvements. Many of these ideas emerged as direct responses to the barriers and challenges described in the previous theme. The current theme, however, focuses on thoughts and reflections aimed at enhancing the method to better meet patients’ needs. It is divided into four subthemes: Technical Improvements, Adaptation of Materials, Development and Revision of the Treatment Program, and Strengths and Opportunities of VRAPT.

#### Subtheme: Technical improvements

There was broad agreement among therapists regarding dissatisfaction with the pulse-monitoring wristband. Despite this, several highlighted the value of measuring biological responses and the importance of a functioning pulse monitor. One therapist stated:

… it would have been really exciting if it had worked, to measure whether they’re actually experiencing tension… or if there’s no tension at all, then we might be dealing with psychopathic traits.

Therapists emphasized that the usability of the equipment needs to be improved and simplified. As previously mentioned, the setup placed high demands on therapists. A recurring suggestion was to better integrate and streamline the equipment’s components. One therapist remarked:

… it could’ve been more integrated, like the pulse monitor and skin conductance, if it could simply indicate high and low.

This quote suggests that the technology should be designed to interpret and present information—such as skin conductance, in a more accessible format. This would reduce workload and enhance usability.

Several therapists also wished for improved graphics and an expanded range of virtual environments and interactions in the VR program. One therapist explained:

… what I noticed was missing was more movement… this limited our ability to carry out certain scenarios the way we had hoped. The same goes for gestures… I would have liked to do more or at least have more options.

They argued that by expanding the range of VR interactions, the experience could be better tailored to individual needs and allow for more dynamic roleplays.

#### Subtheme: Adaptation of materials

Therapists offered numerous suggestions for improving the treatment materials, particularly the manuals and workbooks. One therapist suggested:

… it would’ve been helpful to combine the manual with the workbook, because switching between them is difficult. Some information is in the manual but not in the workbook, so you have to jump between them.

Most suggestions involved adjusting the materials to better fit the patient group by using less text, reducing repetition in homework tasks, and including more visual support. One therapist noted:

… the manual contained a lot of text and repetitive homework, which became a source of irritation for patients… you could have more visual aids and less text.

Another therapist commented on the volume of homework:

… homework is always challenging for most patients… it needs to be better adapted to our population—shorter tasks they can actually manage. Instead of giving them the whole workbook with tons of tasks, just give them two pages for this session.

In short, the workbook and its homework assignments can become overwhelming for patients, and distributing materials per session might be more manageable.

#### Subtheme: Development and revision of the treatment program

Regarding the program’s structure, several therapists emphasized the importance of incorporating functional analyses. Many expressed a desire to get to know the patients better in order to tailor the sessions more effectively. They believed functional analysis would increase the meaningfulness of the treatment. One therapist explained:

… I think we should have paused more… to do behavior analysis together. Then we could better define the treatment goals—what the patient wants to change and why.

Another therapist highlighted how functional analysis could improve roleplay design:

As I mentioned, I’d have liked more time for these behavior analyses—to really ground the roleplays more than I was able to.

Several therapists also suggested adding follow-up sessions and implementing a maintenance plan:

One idea I had for improvement is to include a follow-up session where you go through a plan for maintaining the strategies learned during VRAPT… so they can continue independently.

Another therapist clarified why this is especially important in forensic psychiatry:

… follow-ups, repetition, and continued support are essential. These patients need recurring interventions for the treatment to be sustainable.

#### Subtheme: Strengths and opportunities of VRAPT

Overall, therapists had a positive experience with VRAPT and appreciated its clear CBT-based manual and focus on behavioral interventions. VRAPT was frequently described as a structured and concrete program. One therapist remarked:

I thought it was very good. For me, it was easy to understand. Manageable. The concept was solid, comprehensible. I could translate it into something patients could grasp—that’s what I really appreciated.

VRAPT was also seen as innovative and relevant for future psychiatric care, both in treatment and assessment:

I think VR is probably here to stay in psychiatry—I’m pretty sure of that.

Therapists also noted that the method adds value to existing psychotherapy options. The more treatments available, the greater the chance patients will find something that fits their needs. One therapist said:

… I really believe this can serve a purpose for certain groups who are often missed by traditional treatments.

### Interviews with ward staff

**Table 4:** Summary of results from interviews with ward staff

Main Theme	Subtheme
Experiences of patients in forensic psychiatry	Patients’ everyday lives before VRAPT
	Perceived changes after VRAPT
Experiences of VRAPT in forensic psychiatry	Ward staff’s varying involvement in VRAPT
	Ward staff’s lack of insight into VRAPT
Opportunities with VRAPT	Adapting treatment to the individual
	Additional uses for VRAPT

#### Theme: Experiences of patients in forensic psychiatry

This theme concerns the ward staff’s experiences of the patients they are in close contact with in forensic psychiatry. To gain insight into how the patients were prior to VRAPT, we examine the ward staff’s descriptions and experiences of the patients’ everyday functioning. This provides a perspective for understanding the perceived changes following the initiation of VRAPT treatment. The theme is divided into two subthemes: Patients’ everyday lives before VRAPT and Perceived changes after VRAPT.

#### Subtheme: Patients’ everyday lives before VRAPT

In the interviews, ward staff described various patients before treatment as aggressive and lacking strategies to recognize and manage emotions. Several were also described as closed-off, prone to violence, and self-centered. One staff member stated:

*I would probably describe [them] as one of our most violence-prone patients in the entire clinic*,* with a history of very brutal violence. On the ward*,* [they were] very open about their thoughts of harming people*,* and that was already the case before. [They] would willingly talk about it*,* constantly thought about harming others*,* more or less every day*,* so before*,* it was a lot of violence*,* irritation*,* and easily provoked.*

A self-centered approach to others was described as follows:

*There was no sense of understanding—[they] were very ME-focused*,* like ‘I want this*,*’ [they] would just bulldoze over everyone else.*

While being emotionally closed-off may seem harmless, within forensic psychiatry it can conceal important issues that ought to be addressed:

*[They] are very skilled at not showing what [they’re] thinking*,* but [they] think about hurting people every day. That is something that’s part of [their] diagnosis and [they’re] aware of it. But [they] do talk about it now and then with certain people and try to avoid acting on those thoughts.*

#### Subtheme: Perceived changes after VRAPT

Many staff members found it difficult to report clear changes in levels of aggression after VRAPT. In cases where ward staff perceived changes in patients, these often involved increased self-reflection and more emotional content in conversations:

*Before VRAPT*,* we could spend entire Fridays discussing good movies we’d seen … or what music we liked*,* and so on. We didn’t talk about anything deeper. Then suddenly the patient began returning to thoughts about the crime and why [they] did it*,* whether something was wrong with [them]*,* and there was a lot of reflection. Maybe it triggered more reflection—that’s possible.*

Several staff members observed that patients were better at staying calm in triggering or stressful situations, often using various coping strategies. Improvements in emotional regulation were also noted:

*It’s mainly that they seem calmer. They seem to handle emotions and situations better. That’s how I experience them.*

As with any treatment, staff also noticed an increase in strong emotional expressions in some patients. At the same time, some patients were perceived to have become more open in their demeanor. Other reported changes included greater humility, reduced negativity, and in some cases, a sense of validation and increased meaning in their daily lives.

### Theme: Experiences of VRAPT in forensic psychiatry

This section explores how the staff members who work most closely with the patients on a daily basis perceive their insight into the VRAPT project and treatment method, as well as their own involvement. The theme is divided into two subthemes: Ward staff’s varying involvement in VRAPT and Ward staff’s lack of insight into VRAPT.

#### Subtheme: Ward staff’s varying involvement in VRAPT

While a few staff members had participated in some or all of the VRAPT sessions, the majority stated that they had not been involved at all. Some even mentioned that they were unaware this was an option. However, all staff were assigned the task of conducting weekly patient assessments as part of VRAPT. Most of them believed that their participation would likely have had a positive impact on the treatment process. They reasoned that it would have given them a better overall picture and made it easier to complete the weekly assessments if they had attended the sessions. Several also expressed that increased involvement would have helped them support the patients between sessions more effectively. One interviewee explained how staff involvement could contribute between sessions:

*Absolutely. For example*,* at the start*,* when a plan is made for the patient*,* what they’re going to go through in VR*,* what goals are set*,* is there a short-term goal? A long-term goal? How do we want things to look? But if staff are involved in this*,* then it’s not just that the patient does this on a Monday*,* and then again the next Monday*,* with nothing in between. We have the opportunity to support the patient in between sessions.*

In other words, the ward staff wished to be included in the treatment planning and goal setting in order to better support the patients during the time between sessions.

#### Subtheme: Ward staff’s lack of insight into VRAPT

All ward staff reported that they did not know what treatment goals had been set for the patients. Some stated that they lacked information about the program itself, the patient’s participation, and a general overview of the treatment. One staff member expressed a desire to learn more in order to better support the patient:

*I think it’s an interesting treatment method*,* but since I know so little about it*,* I’d like to learn more so I can help the patient better.*

It was also mentioned that, without this insight into VRAPT, it is difficult to observe any progress, as they do not know what to look for. As one person put it:

*No*,* if I had known more precisely what had been worked on*,* say*,* a patient had these specific issues and that’s what you’ve been targeting*,* none of that has been communicated to me. If I had known they had been practicing something difficult*,* I could have paid more attention to that. But I wasn’t involved*,* so I can’t really say whether it has improved or worsened.*

### Theme: Opportunities with VRAPT

The ward staff identified several opportunities for further development or improvement of VRAPT. This section highlights suggestions that are not yet implemented in the program and is divided into two subthemes: Adapting treatment to the individual and Additional uses for VRAPT.

#### Subtheme: Adapting treatment to the individual

In the interviews, several staff members emphasized how beneficial it would be to further tailor the treatment to each patient’s specific needs. One interviewee stated:

*… I can imagine that here we could really tailor something for each patient. Depending on the patient’s particular issues and the problems one wants to address*,* you could actually customize quite a lot.*

The staff reflected on the potential to expand the number of virtual environments in order to make the treatment and VR role-plays more individualized. Several concrete suggestions for new virtual environments were offered, based on their experience with patients’ difficulties and backgrounds:

*There are people who commit sexual offenses and are triggered by women*,* so maybe you could have a nightclub scenario where a woman rejects you*,* saying ‘I’m not interested’ in a sharp tone that triggers something. There are many moments one could practice.*

#### Subtheme: Additional uses for VRAPT

The staff also shared ideas about possible applications of VRAPT beyond its current use. Suggestions included using the VR technology to train communication and social skills, as these were identified as common challenges among patients:

*Here you have a perfect opportunity to train vocabulary*,* to explain oneself in a positive way. Instead of getting angry when the message isn’t understood and the other person also gets angry. [They] could learn to say something differently*,* and the other person would react differently*,* which would expand vocabulary and the understanding that not everyone thinks the same way. I believe there is potential and room for development. Absolutely.*

They also suggested that continuing the treatment on the ward between sessions could be beneficial. A further idea was to extend the program with real-life practice scenarios as a more advanced stage of training:

*If this is my patient*,* maybe we should test these difficult situations in the VR environment*,* and then*,* based on your recommendation*,* if the patient has succeeded and practiced different difficult scenarios*,* then maybe we should go out into real life and test them there.*

Other staff members proposed additional uses of VR, such as incorporating physical games like boxing or using relaxing virtual environments as a preventive measure for emotional regulation.

## Discussion

The aim of this study was to explore and understand how therapists and staff have experienced VRAPT within the forensic psychiatric ward they work in, in order to contribute to the evaluation of the intervention. To achieve this aim, we sought to answer four research questions. To address the first research question, how therapists and ward staff perceive and experience VRAPT and its implementation within forensic psychiatric care, all interviewees generally expressed a positive attitude toward the project. The use of VR technology in forensic psychiatry was regarded as beneficial and innovative. Therapists particularly appreciated the VR roleplays, though they highlighted several technical difficulties, lack of individualization in the treatment, and challenges associated with low patient motivation. Several members of the ward staff described and problematized their limited insight into the structure and content of VRAPT, while it was also observed that staff involvement in the program varied. In response to the second research question, what changes were perceived in the patients, both therapists and ward staff described similar experiences of increased self-reflection and improved emotional regulation among patients. However, changes in aggression were more difficult to assess, and neither therapists nor staff reported any major differences in perceived levels of patient aggression. The third research question, concerning suggestions for further development of VRAPT, was addressed through a range of concrete proposals from both groups, with a shared emphasis on the need for more individualized treatment. In response to the fourth research question, concerning similarities and differences in the experiences of the two staff groups, no major differences emerged in how they described VRAPT. Their perceptions of the program’s content, impact, and areas for development were largely consistent. However, as several members of the ward staff expressed limited insight into the program, this lack of access may have hindered their ability to provide nuanced or critical reflections on the intervention. Therefore, the absence of differences in their interview responses does not necessarily indicate genuine consensus but may instead reflect varying levels of involvement and understanding of VRAPT.

### Interpretation of the results

Both the revised and first versions of VRAPT, studied in the Netherlands, have officially focused on reactive aggression. One of the inclusion criteria for both the RCT in the Netherlands and the ongoing pilot study in Sweden is “a background of aggression and current problems with reactive aggression” [44, 48]. However, the results of this study show that some therapists noted their patients’ aggressive behavior was characterized by instrumental violence, which the VRAPT method struggled to address. Research on treatment of instrumental aggression is limited, and such aggression is generally considered more difficult to treat due to a lack of motivation, given that this type of aggression does not occur in affect but is instead conscious, goal-directed, and often rewarding in nature [55–57].

The presence of some patients displaying instrumental aggression in the current pilot study may be explained by VRAPT’s theoretical foundation, the GAM, which does not treat reactive and instrumental aggression as separate categories but rather adopts a multidimensional perspective on aggression [23]. Aggressive behaviors often contain elements of both types, which may overlap or be present at different times in a person’s life. GAM was developed to account for such complex aggression scenarios [23, 48]. An essential area to address when tackling the issue of aggression in forensic settings, particularly in forensic psychiatry, is the recognition that aggression is a complex and multidimensional phenomenon. The categorization of aggression into reactive, instrumental, and psychotic is well established [16, 58]. At the same time, in clinical practice, it is frequently observed that patients may present with various combinations of psychotic, reactive, and instrumental aggression, with numerous possible variations among these forms. Furthermore, individuals with predominantly reactive aggression often struggle with impulsivity, which, as highlighted in therapists’ interviews, can make it difficult to maintain focus and motivation in a treatment described as repetitive and at times monotonous. In some cases, patients were perceived as not fitting the treatment, whereas it might be more appropriate to adapt the treatment to fit the patients. Both therapists and staff emphasized the importance of tailoring interventions to individual needs. Many forensic psychiatric patients have neurodevelopmental disorders, and would likely benefit from shorter, simpler materials with visual support to aid comprehension. A challenge with including patients who predominantly exhibit instrumental aggression is that they may conceal their reactive aggression from therapists. For example, admitting to any internal aggression, while being key to treatment, could potentially carry a risk of prolonging a sentence at the ward. In contrast, patients who predominantly exhibit psychotic aggression are generally well managed with antipsychotic medication, which often reduces aggression related to psychotic symptoms. Acknowledging this complexity is crucial for supporting the development of interventions such as VRAPT, or other treatments targeting aggression through VR-assisted approaches [16].

One striking finding from the interviews was that both therapists and staff sometimes expressed that they had not observed changes in patients following VRAPT, while simultaneously describing concrete positive developments such as improved communication and increased self-awareness. This discrepancy may suggest that VRAPT fosters changes in domains other than those it primarily targets, namely, aggression. It is possible that these changes are the first steps to the primary target of reducing aggression. The staff’s statements align with previous findings from the RCT conducted in the Netherlands, where secondary outcomes such as anger management, impulsivity, and hostility improved, while the primary outcome of aggressive behavior showed no statistically significant change [44]. Violent incidents still occurred among patients during and after VRAPT treatment. According to GAM, aggressive response patterns become automated through repeated prior experiences. Changing aggressive individuals thus requires reshaping their interpretations of the world and their learned behavioral repertoires, something that becomes increasingly difficult with age [23]. The revised version of VRAPT places greater emphasis on emotional recognition and regulation, as GAM posits that increased insight and cognitive control can help individuals better manage aggressive impulses [45]. This may help explain staff observations of certain improvements despite persistent aggression. It should be noted that it is not possible to determine whether the violent incidents were related to VRAPT. Ward staff described difficulties in evaluating patients due to external circumstances on the wards, medication, comorbid diagnoses, and limited insight into the VRAPT program. While some staff perceived changes in patients and others did not, it is important to emphasize that “change” in this study refers solely to the subjective perceptions of therapists and staff, not to objectively measurable change. As such, our study does not allow for general conclusions about whether patients improve or deteriorate as a result of the intervention.

Ward staff expressed a desire to be more involved in VRAPT, while therapists reported a need for more time to conduct functional analyses to better understand their patients. A possible solution to both issues is to draw on ward staff’s knowledge of the patients when conducting functional analyses. Both therapists and ward staff agreed that follow-up would have benefited the patients. In this context as well, the staff could have contributed to planning and implementation on the wards. There are multiple benefits to interprofessional collaboration, both for the patients and for the organization. From an organizational standpoint, it is economically advantageous when knowledge is shared rather than isolated within professions. For the patients, a multidisciplinary approach increases the chances of identifying and addressing issues from multiple angles.

Several therapists stated that their patients needed space for conversations outside the formal structure of VRAPT. One possible solution could be to incorporate time for these types of sessions, where patients can process thoughts and emotions that arise during treatment. This might help them work through psychological challenges without disrupting the structured format of VRAPT. This also appears to be an ethical dilemma for therapists: whether to meet the patient’s individual needs or adhere strictly to the treatment manual, even when it may not be entirely appropriate for the situation. Clearer guidelines on how to handle such cases could offer therapists better support in their clinical practice.

The results of this study are largely consistent with previous research on patients’ experiences of VRAPT within Swedish forensic psychiatry [48]. In both studies, patients and therapists reported positive experiences with the VR role-plays, which were perceived as novel and engaging. However, frustration related to technical limitations was evident across both datasets. For patients, this frustration primarily concerned the limited quality of graphics and restricted interaction possibilities, which diminished the sense of immersion [48]. Therapists, in contrast, highlighted technical issues that interfered with the continuity and flow of treatment sessions. Importantly, patients described increased self-awareness and enhanced capacity for reflection, experiences that were also observed by therapists and ward staff in the current study. Both studies emphasized the need for greater individual adaptation of the intervention and suggested the inclusion of structured follow-up components to strengthen its clinical utility. These findings are further supported by a related pilot study, which showed that participants generally received the VR experience, including role-play exercises, positively, although some reported low motivation and found parts of the treatment to be repetitive and monotonous [59]. While VR technology was regarded as a promising tool, the study also pointed to technical constraints and underscored the importance of tailoring the intervention in both content and format, particularly for patients with neurodevelopmental disorders. Collectively, these results highlight the importance of integrating clinical and technological perspectives in the ongoing development of VRAPT and underscore the need for continued research into how VR-assisted interventions can be effectively adapted and implemented in forensic settings.

### Strengths and limitations

This study contributes new perspectives by including both therapists’ and ward staff’s experiences of VRAPT within Swedish forensic psychiatry, something that had not been previously examined. It also adds new knowledge about staff’s perception of limited insight into VRAPT, which was not addressed in the earlier patient-focused study. Since staff can contribute to the project and treatment in various ways, as previously described, this evaluation is valuable for the future development of the method. The therapists’ statements, such as the high demands for multitasking that complicate the treatment work in various ways, are also valuable for the continued development of both the treatment method and the manual.

We view the circular, rather than linear, nature of our working process as a methodological strength. This iterative approach facilitated a deeper engagement with the material and enabled a more nuanced and comprehensive analysis. By integrating the research questions, data analysis, and theoretical framework in a dynamic and reciprocal manner, we were able to develop the study holistically rather than in compartmentalized phases. This reflects the iterative nature of qualitative research, in which emerging interpretations and insights continuously inform further reflection and analysis. Another notable strength of the study lies in the inclusion of two professional groups, therapists and ward staff, which enabled methodological triangulation. Comparing the perspectives of these two groups yielded a coherent and consistent picture of VRAPT, thereby enhancing the study’s credibility and increasing the transferability of the findings.

Our study addresses a knowledge gap and contributes practical value by improving safety and rehabilitation for both staff and patients. As previously mentioned, the evidence base in forensic psychiatry is insufficient, so we find it meaningful that our study may help strengthen the foundation for evidence-based aggression treatments in a Swedish context. The study also provides new insights from both therapists and ward staff, something not previously explored in evaluations of VRAPT.

The ward staff’s limited insight into the intervention may have affected their ability to provide more in-depth descriptions of VRAPT, which represents a limitation of the study. At the same time, their experience of lacking insight is, in itself, important and worth highlighting. Greater involvement might have enabled more comprehensive responses, which may be of interest in future research. The forensic psychiatric setting may limit the generalizability of the findings, as it involves detained patients with complex comorbid diagnoses and low motivation, factors that influence the therapeutic alliance and other treatment processes.

Validity in qualitative research is assessed based on credibility, transferability, and meaningfulness, among other factors. The credibility of a study is primarily influenced by the method of analysis, where no information should be added or omitted, but rather grounded in the participants’ perspectives. The credibility of this study is strengthened by our efforts to represent the experiences of the therapists and staff as expressed in the transcripts [50]. Our themes are supported by quotes, allowing the reader to assess the interpretation for themselves. We also maintained a consistently reflective approach, continually considering our own preconceptions and how they might influence data analysis. As we are two authors rather than one, we were able to compare and discuss individual interpretations to arrive at a more coherent and nuanced understanding. Any deviations from standard procedures, such as conducting two rounds of analysis instead of one, have been thoroughly documented in the methods section. Both transparency and reflection are key contributions to qualitative validity. In a qualitative context, transferability does not concern quantity or frequency but rather methodological rigor and sampling, how the research contributes to the population and topic being studied [50]. Contextual information about the study’s participants, setting, and environment has been described in detail. Although the results of this study are specific to the given group and context, they may still be relevant to similar contexts of aggression, such as domestic violence or youth with impaired impulse control.

### Ethical challenges

The findings must be interpreted in light of the ethical complexity inherent in forensic psychiatric settings. The distinct roles of ward staff and therapists influenced their perspectives on the intervention, with ward staff reflecting more directly on patient experiences and therapists focusing on treatment structure and therapeutic potential. This division of roles helped to minimize the classic dual-role conflict, where the clinician simultaneously assumes responsibility for care and research [2, 60, 61]. However, ethical tensions persist, particularly regarding the risk of viewing patients as subjects rather than individuals, especially when novel interventions such as VRAPT are introduced in secure environments. The ethical imperative, therefore, is not only to protect participants from harm but also to ensure that their rights, autonomy, and dignity are preserved. Future implementations should consider establishing additional safeguards, such as involving patient representatives in the evaluation process and maintaining clear boundaries between clinical duties and research involvement.

### Future research

The findings of this study provide a foundation for identifying areas that require further development, particularly those aspects that proved challenging during implementation. Based on the results, VRAPT may benefit from enhancements in key areas such as personalization, technical functionality, and strategies to strengthen patient engagement. These insights underscore the importance of future studies focused on advancing and refining VR-assisted aggression treatment. Further research could investigate whether VRAPT is particularly effective in addressing specific aggression-related outcomes, or whether its impact extends to broader psychological constructs such as emotion regulation, metacognition, and self-awareness. Evaluating the effects of VRAPT over time, both immediately after treatment and at follow-up intervals, such as six months post-intervention, may offer important insights into the trajectory of change. This is especially relevant in forensic psychiatric settings, since patients may experience an initial increase in distress before meaningful therapeutic gains become evident. Given that many individuals remain in forensic care for extended periods, longitudinal research examining the development of aggression and related psychological processes within the context of VR-assisted interventions would be especially valuable. VRAPT has already been evaluated in a prison setting in Sweden [49, 62], demonstrating its potential within institutionalized populations. However, it would also be important to examine its application among individuals with aggression-related problems who are not institutionalized. Such research could help assess the generalizability of the method and its relevance for a broader range of clinical and community settings.

## Conclusion

This study explored the experiences of therapists and ward staff with virtual reality-assisted aggression therapy in a maximum-security forensic psychiatric clinic. Thematic analysis of interviews with therapists resulted in five main themes: When Treatment Clashes with Low Motivation, The Importance of Roleplay in VRAPT, Experiences of Patient Development, Obstacles and Problems Hindering the Treatment, and Ideas for Further Development. The results indicate that VR roleplay was perceived as central and was the most appreciated element of VRAPT among therapists. However, technical difficulties posed challenges during treatment. When working with patients with low motivation and cognitive difficulties, the treatment manual was at times experienced as overwhelming and repetitive. Increased individualization of the treatment was therefore a recurring suggestion. Thematic analysis of interviews with ward staff resulted in three main themes: Experiences of Patients in Forensic Psychiatry, Experiences of VRAPT in Forensic Psychiatry, and Noted Opportunities with VRAPT. The findings provide a nuanced understanding of the challenges in evaluating aggression. External circumstances, violent incidents, medication, comorbid diagnoses, and ward staff’s limited insight into VRAPT make it difficult to determine which changes are attributable to the treatment. Nevertheless, increased self-confidence and self-reflection were observed, aligning with the therapists’ observations. The ward staff emphasized the need for individualization and expressed a desire to be more involved in order to better support patients with their homework, which was rarely completed. This study has contributed new and valuable in-depth knowledge about VRAPT from a forensic psychiatric staff perspective.

## Data Availability

The datasets generated and analyzed during the current study are not publicly available due to confidentiality agreements but are available from the corresponding author on reasonable request.
